# Significant Succession of Intestinal Bacterial Community and Function During the Initial 72 Hours of Acute Pancreatitis in Rats

**DOI:** 10.3389/fcimb.2022.808991

**Published:** 2022-04-29

**Authors:** Jinbo Liu, Ming Luo, Shu Qin, Bo Li, Lin Huang, Xianming Xia

**Affiliations:** ^1^Department of Hepatobiliary Surgery, The Affiliated Hospital of Southwest Medical University, Luzhou, China; ^2^Academician (Expert) Workstation of Sichuan Province, The Affiliated Hospital of Southwest Medical University, Luzhou, China; ^3^Clinical Research Institute, The Affiliated Hospital of Southwest Medical University, Luzhou, China

**Keywords:** 16S rRNA gene, acute pancreatitis, cluster of orthologous genes, *Firmicutes/Bacteroidetes* ratio, intestinal microbiota, Kyoto Encyclopedia of Genes and Genomes

## Abstract

Acute pancreatitis (AP) is followed by structural and functional changes in the intestine, resulting from microbiome dysbiosis. However, it remains unclear how gut microbiome changes within the initial 72h of onset. In this study, severe acute pancreatitis (SAP), mild acute pancreatitis (MAP), and sham operation (SO) were replicated in rat models. 16S ribosomal RNA gene sequencing was used to explore the gut bacteria community. The predicted Cluster of Orthologous Genes (COG) and Kyoto Encyclopedia of Genes and Genomes (KEGG) metabolic pathways were associated with the 16S rRNA profiles. Compared to the SO group, significant community succession was found during the initial 72h in AP group. At 72 h after AP induction, the *Firmicutes/Bacteroidetes* (F/B) ratios were significantly different, with the highest ratio in SAP group and the lowest in MAP group. *Lactobacillus* was the most abundant genus, but it nearly disappeared in SAP rats at 72 h. *Clostridiaceae* 1 and *Clostridium sensu stricto* 1 were significantly enriched in AP group. *Bacteroidales* S24-7 and *Bacteroidales S24-7 group norank* were enriched in MAP group, while *Collinsella*, *Morganella*, and *Blautia* were enriched in SAP group. *Lactobacillus* was significantly correlated with nine COGs. Nine COGs showed significant differences between AP group and SO group. Moreover, four COGs showed significant differences between the MAP and SAP groups. KEGG Level_3 pathways propanoate metabolism (Ko00640) in AP group was significantly higher than that in SO group. The aspartate‒ammonia ligase and four KEGG orthology terms of the AP group were lower than that in the SO group, respectively. All these results suggest that the intestinal bacterial community structure and function was changed during the initial 72h in AP rats. The intestinal F/B ratio and the relative abundance of *Lactobacillus* could be potential markers for early diagnosis of MAP and SAP. The genus *Clostridium sensu stricto 1* was the most enriched genus in AP, and may be an important marker for AP.

## Introduction

Acute pancreatitis (AP) is a prevalent gastrointestinal disorder, which requires acute hospitalisation and has a rising incidence (van Dijk et al., 2017; Li et al., 2020). Mortality rates of it range from > 20% in the highest-risk group to < 1% in the lowest-risk group (Wu et al., 2008). Infected pancreatic necrosis is one of the most severe complications of AP (Susak et al., 2021). Bacterial infection of the pancreatic/peri-pancreatic tissue results mainly from intestinal bacterial translocation (Fritz et al., 2010). Gut translocation of bacteria has been reported in animal models and clinical trials, especially in severe acute pancreatitis (SAP) (Liu et al., 2019). Infectious complications are a leading cause of death for patients with SAP (Li et al., 2018). Moreover, SAP can cause systemic endotoxemia, and SAP patients exhibit intestinal permeability which was shown to correlate with increased early endotoxemia (Schietroma et al., 2016). Hence, the succession of intestinal bacteria has become a research focus in the past two decades, as the key bacteria involved may become potential markers for the diagnosis, and even targets for treatment of AP.

Gut microbiome is considered as a crucial regulator of human health. Alteration of gut microbiome has been reported in AP, and it probably contributes to the severity of disease (Zhu et al., 2021). Bacterial translocation in blood from patients with SAP has been reported (Wen et al., 2017). In serum from AP patients, *Citrobacter freundii* and *Pseudomonas aeruginosa* have been detected (de Madaria et al., 2005). *Clostridium perfringens* is also an important biomarker of necrotizing pancreatitis (Biswas et al., 2017). The opportunistic pathogens translocated in AP patients are mainly derived from the intestine, including *Escherichia coli*, *Shigella flexneri*, *Enterobacteriaceae bacterium*, *Acinetobacter lwoffii*, *Bacillus coagulans*, and *Enterococcus faecium (* Li et al., 2013). These bacteria are frequently altered in MAP patients, and significantly correlated with inflammation in those with SAP, indicating that AP progression may be affected by the intestinal microbiota composition (Tan et al., 2015). In addition, antibiotics were found to induce imbalances in the microbiota of the small intestine and bacterial translocation in a mouse model of SAP (Soares et al., 2017).

It has also been reported that the microbiome can translocate to multiple sites in perioperative pancreaticoduodenectomy patients (Rogers et al., 2017). The specific composition of the gut microbiome is associated with higher rates of postoperative complications after pancreatic surgery (Schmitt et al., 2019). Regardless of the disease state, bacterial DNA profiles in the pancreas are similar to those in the duodenal tissue of the same subjects, suggesting that bacteria may migrate from the gut into the pancreas (del Castillo et al., 2019). Furthermore, gut microbiome dysbiosis worsens the severity of AP in humans and in mouse models (Zhu et al., 2019). Additionally, impaired exocrine pancreatic function was found to be related to changes in intestinal microbiome composition and diversity (Frost et al., 2019).

Thus, the intestinal microbiome is an important regulator of AP, and microbiome composition may be a potential biomarker for AP pathogenesis and an indicator of pancreatic function. However, previous studies have provided different descriptions of the characteristic bacterial genus involved in animals and humans with AP. Most of these studies have focused on a single time point in AP animal models, with a long interval (within 7 days) of onset of AP patients (Tan et al., 2015; Zhu et al., 2019), some research based on the blood detection of bacterial translocation (de Madaria et al., 2005; Li et al., 2013; Wen et al., 2017; Li et al., 2018). The successive changes in intestinal bacterial communities during the initial disease stage, which is the key period for disease control, is still unclear. In this study, we set up acute pancreatitis models *via* rat. Then we explored the bacterial community succession and associated functional changes during the initial stages of acute pancreatitis, using next generation sequencing. And we found significant succession of intestinal microbiota and their predicted function during the initial stages of MAP and SAP in rat models.

## Materials And Methods

### Experimental Design and Sampling

The experimental program was approved by the Experimental Animal Ethics Review of Southwest Medical University (approval number: 20180517002). AP was induced following the protocol described by Yao et al. (2020).

Healthy adult male Sprague‒Dawley rats from the Experimental Animal Center of Southwest Medical University (weighing 300 ± 20 g) were used in this study. Rats were kept under a light–dark cycle (every 12 h) at a constant temperature of 24°C. Water and rat chow were freely accessible. Each cage housed five rats.

Rats were divided into MAP, SAP, and sham-operated (SO) groups, with 20 rats in each group. After 1 week of adaptation, MAP and SAP were induced in the rats by retrograde injection of 0.5% and 4% sodium taurocholate solution, respectively, at a volume of 1 mL/kg, into the biliopancreatic duct, the speed of administration was controlled by a micro-infusion pump at 0.1 mL/min. Rats in the SO group were injected with saline rather than with sodium taurocholate.

At each time point (24, 48, and 72 h), rats were sacrificed, with five rats per group at each time point. The pancreas, distal small intestines, and fresh feces were collected. Fecal material was immediately snap-frozen in liquid nitrogen and stored at -80°C. Histological examination of the pancreas and intestinal tissues was performed according to the protocol described by Zhao et al. (2020).

### Fecal Nucleic Acid (DNA) Extraction and 16S Ribosomal RNA (rRNA) Gene Amplification

The E.Z.N.A.^®^ soil DNA Kit (Omega Bio-tek, Norcross, GA, USA) was used for microbial DNA extraction from fecal samples, following the manufacturer’s protocols. From each group, three representative samples were selected for each time point. DNA purity and content were determined by NanoDrop 2000 UV-vis spectrophotometer (Thermo Scientific, Wilmington, NC, USA), and 1% agarose gel electrophoresis was also used to evaluate DNA quality.

Primers 338F (5’-ACT CCT ACG GGA GGC AGC AG-3’) and 806R (5’-GGA CTA CHV GGG TWT CTA AT-3’) were selected for bacterial 16S rRNA gene amplification, focusing on the V3‒V4 hypervariable regions, using a thermocycler (GeneAmp 9700, ABI, Foster City, CA, USA). PCR was performed using the following thermocycling profile: 95°C denaturation for 3 minutes; followed by 27 cycles consisting of 30 seconds at 95°C, annealing for 30 seconds at 55°C, and elongation for 45 seconds at 72°C, and a final extension of 10 minutes at 72°C. Triplicate reactions were performed; total reaction volumes were 20 μL, with mixtures containing 5 × FastPfu Buffer 4 μL (Abbexa Ltd., Cambridge, UK), 2.5 mM dNTPs 2 μL, each primer 0.8 μL (5 μM), FastPfu polymerase 0.4 μL (Cambridge, UK), and template DNA 10 ng. Separation by electrophoresis in 2% agarose gel was used for PCR product extraction, and products were purified using the AxyPrep DNA Gel Extraction Kit (Axygen Biosciences, Union City, CA, USA) and quantified using QuantiFluor™-ST (Promega, Madison, WI, USA) following the manufacturer’s protocol.

### Next Generation Sequencing, Sequence Data Processing, and Functional Annotation

Next generation sequencing was performed on the Illumina MiSeq sequencing platform using PE300 chemical at Majorbio Bio-Pharm Technology Co., Ltd. (Shanghai, China) under standard protocols. Trimmomatic (USADELLAB.org) was used as a raw fastq files quality filter, then reads with an average quality score of < 20 over a 50-bp sliding window and a sequence overlap longer than 10 bps with no more than a 2 bp mismatch were merged by FLASH (https://ccb.jhu.edu/software/FLASH/). Sample sequences were separated according to barcodes and primers, and reads which contained ambiguous bases were removed.

Operational taxonomic units (OTUs) were clustered, using a 97% similarity cutoff, using UPARSE (version 7.1 http://drive5.com/uparse/). Taxonomy was analyzed using the RDP Classifier algorithm (http://rdp.cme.msu.edu/) against the Silva (SSU128) 16S rRNA database, using a confidence threshold of 70%.

Phylogenetic Investigation of Communities by Reconstruction of Unobserved States (PICRUSt; http://picrust.github.io/picrust/) was used for gut microbiome functional annotation (Douglas et al., 2018), with an estimated accuracy of 0.8. The bacterial gene content was predicted for each individual animal.

### Statistical Analysis

In order to illustrate the variation of clustered bacterial community composition, a non-metric multidimensional scaling (NMDS) ordination was conducted using Vegan package of software R. In addition, analysis of similarity (ANOSIM) and partial least-squares discriminant analysis (PLS-DA) were performed to contrast bacterial composition between samples. Statistically significantly different biomarkers between groups were identified by linear discriminant analysis (LDA), coupled with effect size measurements (LEfSe) (Segata et al., 2011). The differences in the relative abundance, predicted function (COGs, KEGG), and correlation analysis between the COGs and the relative genus abundance were calculated among three groups using SPSS (IBM SPSS Inc., Armonk, NY, USA).

## Results

### Morphological Changes Into the Pancreas and Intestine

**Figure 1:**
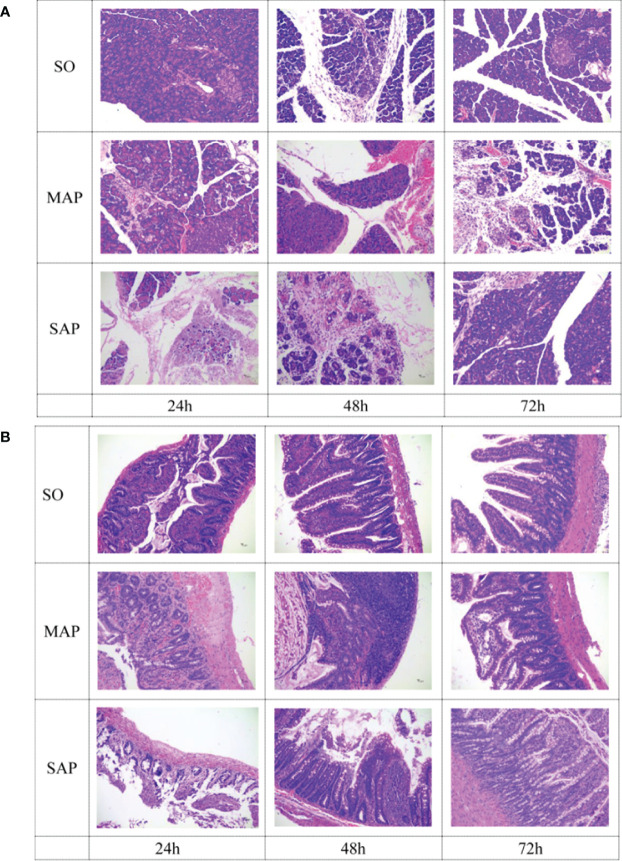
Histological examination of the pancreas **(A)** and small intestine **(B)** samples taken at three time points. SO, Sham operation; MAP, Mild acute pancreatitis; and SAP, Severe acute pancreatitis. Magnification: 200×.

Histopathological changes in pancreas were mainly characterized by varying degrees of acinar cell necrosis and leukocyte infiltration in MAP group and SAP group. In addition, more interstitial edema was found in SAP group than that in MAP group (**Figure 1A**). Similarly, histopathological changes in intestine were characterized by cell necrosis in lamina propria of mucosa, atrophy of intestinal gland, destruction of villi, and inflammatory cell infiltration in MAP and SAP groups (**Figure 1B**). Severe pancreas and intestine damage at three time points could be obviously found in MAP and SAP groups, compared to SO group.

### Operational Taxonomic Units in AP Rat Intestinal Feces

In total, 1,091,966 high-quality sequences were generated for 27 samples, with each sample yielding 36,218 to 47,579 sequences. Minimal sequences were used to equalize the sequence depth of each sample and clustering. In total, 768 OTUs were obtained. Good’s coverage was 99.78 ± 0.01% (mean ± standard error). According to Venn analysis, 619 OTUs were shared among the three groups, accounting for 80.6% of all OTUs. The SO and MAP groups shared 676 OTUs, accounting for 88% of all OTUs. The SO and SAP groups shared 648 OTUs, accounting for 84.38% of all OTUs. The MAP and SAP groups shared 687 OTUs, accounting for 89.45% of all OTUs. From 24 h to 72 h, a clear succession in microbiota communities was observed in the SO, MAP, and SAP groups (**Figure S1**). This result indicated that the bacterial community structures in the MAP and SAP rat intestinal feces were more similar than those in the SO group.

### Bacterial Community Composition in Samples

**Figure 2:**
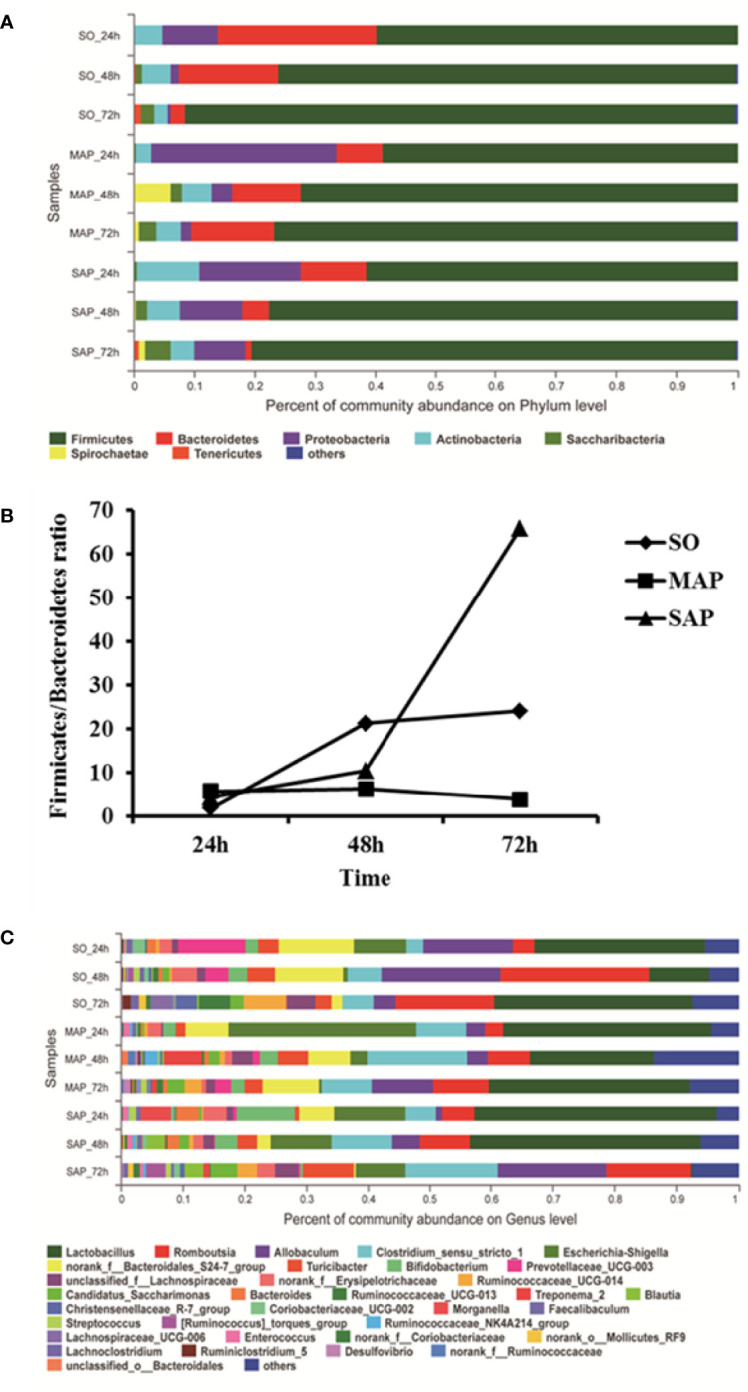
Microbial community composition in fecal samples taken at three time points. **(A)** For each sample, the bar width of each phylum indicates the relative abundance of that phylum in the sample. **(B)** The *Firmicutes/Bacteroidetes* ratio of the three groups. **(C)** For each sample, the bar width of each genus indicates the relative abundance of that genus in the sample. SO, Sham operation; MAP, Mild acute pancreatitis; SAP, Severe acute pancreatitis.

At the phylum level, bacterial community composition is shown in a bar plot (**Figure 2A**). The most dominant phylum was *Firmicutes*. Its relative abundance was above 50% with a consistently increasing trend from 24 h to 72 h in all groups. *Bacteroidetes* was the second abundant phylum. From 24 h to 72 h, its relative abundance decreased in both SO group and SAP group, but increased in MAP group. *Proteobacteria* was the third abundant group, and its relative abundance declined from 24 h to 72 h in all three groups. The *Firmicutes/Bacteroidetes* ratio (F/B ratio) was significantly changed from 24 h to 72 h in the three groups. It was the highest in the SAP group and lowest in the MAP group at 72 h (*P* < 0.05) (**Figure 2B**). These results indicated that the F/B ratio may be a useful marker for distinguishing among the three groups, particularly at 72 h after AP induction.

At the genus level, bacterial community composition is shown in a bar plot (**Figure 2C**). The dominant genus was *Lactobacillus*, with an average relative abundance of over 25%. From 24 h to 48 h, the relative abundance of *Lactobacillus* decreased in SO group and MAP group; from 48 h to 72 h, it was restored to a similar level with that observed at 24 h. In SAP group, levels of *Lactobacillus* remained relatively stable from 24 h to 48 h, after which there was a sudden decrease at 72 h. *Romboutsia* was the second abundant genus. Its relative abundance rapidly rose from 24 h to 48 h, then decreased from 48h to 72 h in SO group. But it increased from 24 h to 72 h in MAP and SAP groups. *Allobaculum* was the third abundant genus, showing a rapid rise from 24 h to 48 h, and a decrease thereafter from 48 h to 72 h in SO group and MAP group, a linear increase was observed from 24 h to 72 h in SAP group. *Clostridium sensu stricto* 1 was the fourth abundant species, with a succession trend similar to that of *Allobaculum*. *Escherichia*-*Shigella* was the fifth abundant group. Its relative abundance declined rapidly from 24 h to 48 h, until it approached zero at 72 h in the SO group and MAP group. Its relative abundance decreased from 24 h to 72 h in SAP group, but remained above 8% at 72 h. The *Bacteroidales* S24-7 group was the sixth abundant group. Its relative abundance remained steady in the MAP group, decreased from 48 h to 72 h in SO group, and decreased from 24 h to 72 h in the SAP group, but remained above 6% in all samples. Spearman’s correlation analysis showed that, the relative abundance of *Lactobacillus* and *Allobaculum* had significant negative correlations; the relative abundance of *Romboutsia* and *Clostridium sensu stricto* 1 had significant positive correlations in total (*P* < 0.05).

### Bacterial Community Beta-Diversity in AP Rats

**Figure 3:**
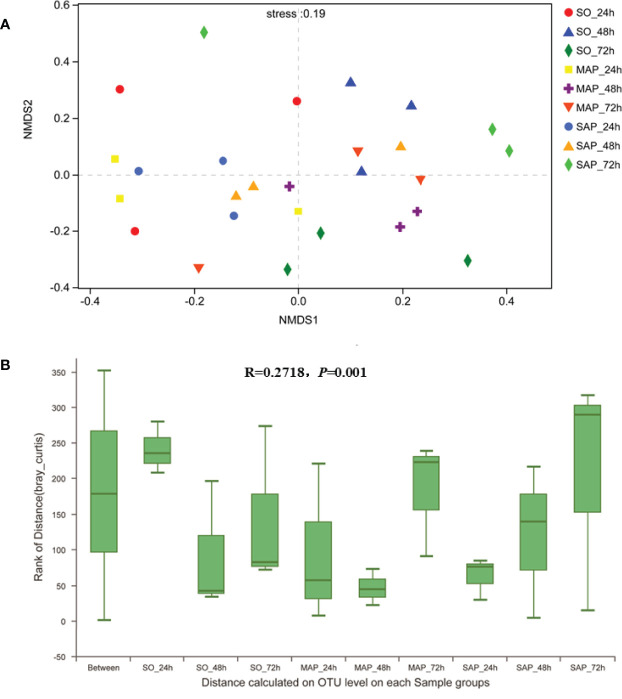
NMDS and ANOSIM analyses of bacterial community composition. **(A)** The different colors in the NMDS analysis represent different groups. **(B)** ANOSIM analysis of bacterial community composition of each sample group. SO, Sham operation; MAP, Mild acute pancreatitis; SAP, Severe acute pancreatitis; NMDS, non-metric multidimensional scaling; ANOSIM, analysis of similarity.

**Figure 4:**
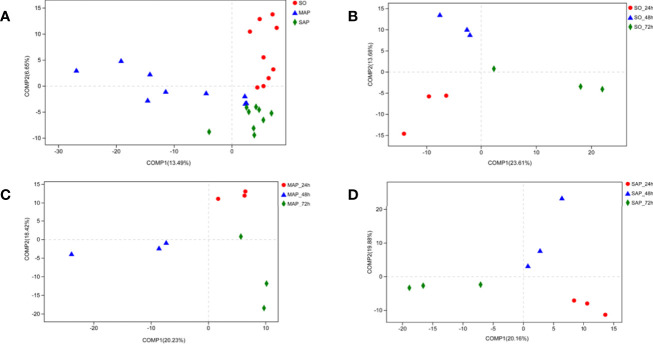
Bacterial community beta-diversity by PLS-DA analyses in fecal samples. **(A)** The different colors in the PLS-DA analysis represent different groups. **(B–D)** PLS-DA analysis demonstrates the difference in the same group at three time points for fecal samples of SO, MAP, and SAP rats. SO, Sham operation; MAP, Mild acute pancreatitis and SAP, Severe acute pancreatitis; PLS-DA, partial least squares discriminant analysis.

NMDS analysis showed that the three groups were not well dispersed with respect to one another (**Figure 3A**). Gut microbiota patterns showed significant differences among the different groups of rats as determined by ANOSIM analysis (R = 0.2718, *P* = 0.001) (**Figure 3B**). The composition of intestinal bacterial communities differed among the groups based on PSL-DA analysis, and differences were also found within each group at the three time points (**Figure 4**).

**Figure 5:**
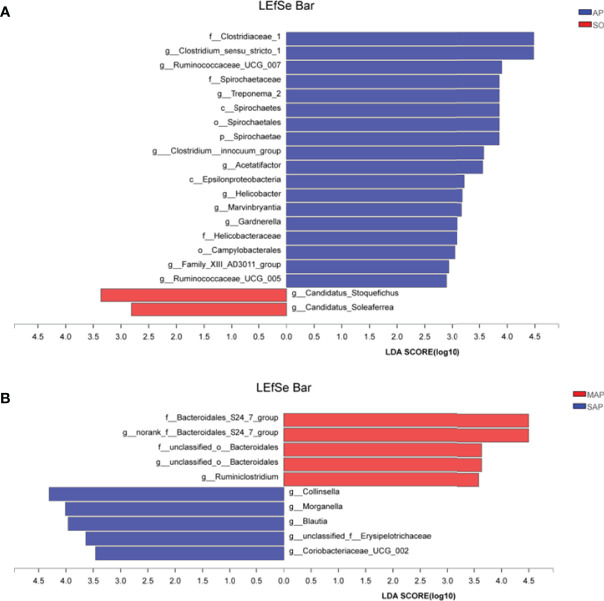
LDA Effect Size (LEfSe) algorithm was applied, on phylum to genus level OTU tables to determine taxa, which is best characterized as each biological class. **(A)** The difference between samples of AP to SO rat. **(B)** The difference between the samples of MAP to SAP rats. SO, Sham operation; MAP, Mild acute pancreatitis; SAP, Severe acute pancreatitis; LDA, linear discriminant analysis. AP indicates MAP plus SAP.

We used LEfSe analysis (LDA threshold of 2) to screen bacterial communities with differences in SO and AP rats fecal samples (**Figure 5**). Compared to the SO group, the family *Clostridiaceae* 1, and the genus *Clostridium sensu stricto* 1 were significantly enriched in the AP (SAP + MAP) group (*P* < 0.05). Between the MAP and SAP groups, the *Bacteroidales* S24-7 group and the genus *Bacteroidales* S24-7 group norank were enriched in MAP group (*P* < 0.05). *Collinsella*, *Morganella*, and *Blautia* were enriched in the SAP group (*P* < 0.05). These results suggested that AP group had a significantly different bacterial community structure compared with SO group. Meanwhile, differences were also found between MAP group and SAP group.

### Prediction of Bacterial Phenotypes

**Figure 6:**
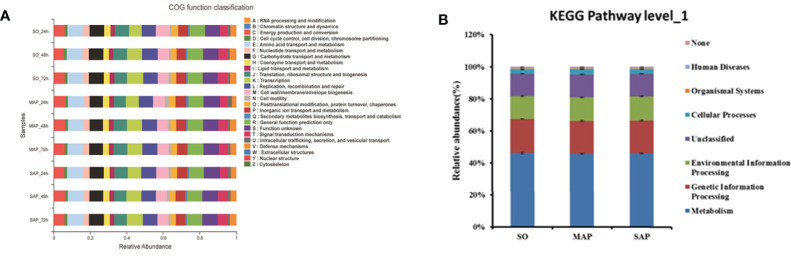
The function predicted for 16S rRNA by PICRUSt. **(A)** For COGs in three groups at three time points. **(B)** For KEGG pathway level_1 in three groups. SO, Sham operation; MAP, Mild acute pancreatitis and SAP, Severe acute pancreatitis; PICRUSt, Phylogenetic Investigation of Communities by Reconstruction of Unobserved States (PICRUSt); KEGG, Kyoto Encyclopedia of Genes and Genomes. Data are shown as mean ± standard error.

Eleven abundant COG functions (relative abundance ≥ 5%) were identified. The most abundant was amino acid transport and metabolism (E) (8.5%), followed by general function prediction only (R); function unknown (S); carbohydrate transport and metabolism (G); replication, recombination, and repair (L); translation, ribosomal structure, and biogenesis (J); cell wall/membrane/envelope biogenesis (M); transcription (K); energy production and conversion (C); inorganic ion transport and metabolism (P); and signal transduction mechanisms (T) (**Figure 6A**). The sequence of the relative proportion of these COGs (from high to low) differed between SO group and AP group.

The 11 abundant functions changed successively from 24 to 72 h in the three groups (**Figure S2**). Among them, R, K, and T increased in all three groups, while M and P decreased in all three groups. At 72 h, K was significantly lower, and J was significantly higher in the MAP than in the SAP group (*P* < 0.05). These results show that the main microbial functions were markedly different among the three groups.

Using KEGG database analysis, we identified seven Level_1 pathways (**Figure 6B**). Metabolism was the most abundant (46%), followed by genetic information processing, environmental information, processing, unclassified cellular processes; organismal systems; and human diseases. These showed similar relative abundances among the three groups.

### Correlation Analysis Between Genus and COG Function

The genera showing a high correlation with COG function are listed in Tables S1‒S2. Thirteen genera were significantly correlated with particular COG functions. The dominant genus, *Lactobacillus*, was totally correlated to nine COGs. It was negatively correlated with defense mechanisms (V) and cell cycle control, and cell division and chromosome partitioning (D), and positively correlated with secondary metabolite biosynthesis, transport, and catabolism (Q), and cell motility (N) (*P* < 0.01). *Romboutsia* was totally correlated to six COGS; it was negatively correlated with P, posttranslational modification, protein turnover, chaperones (O), and U; and positively correlated with K, V, and D (*P* < 0.01). *Allobaculum* was negatively correlated with I and Q (*P* < 0.01). *Escherichia-Shigella* was positively correlated with P (*P* < 0.01). Unclassified *Lachnospiraceae* was totally correlated with four COGS; it was negatively correlated with O and M, and positively correlated with K and T (*P* < 0.01). *Turicibacter* was totally correlated to six COGs; it was significantly correlated with R (*P* < 0.01). *Prevotellaceae* UCG-003 was totally correlated to five COGs; it was positively correlated with J and negatively correlated with N (*P* < 0.01). *Candidatus Saccharimonas* was totally correlated to five COGs; it was negatively correlated with M and O, and positively correlated with T (*P* < 0.01). *Ruminococcaceae* UCG-014 was totally correlated to three COGs; it was negatively correlated with M (*P* < 0.01). *Clostridium sensu stricto 1* was negatively correlated with F (*P* < 0.01). These results showed that *Lactobacillus* was correlated with most COGs, compared to other genus, suggesting that the *Lactobacillus* may play an important role in keeping health intestinal community structures.

### Differences of Predicted Function Among the Three Groups

**Figure 7:**
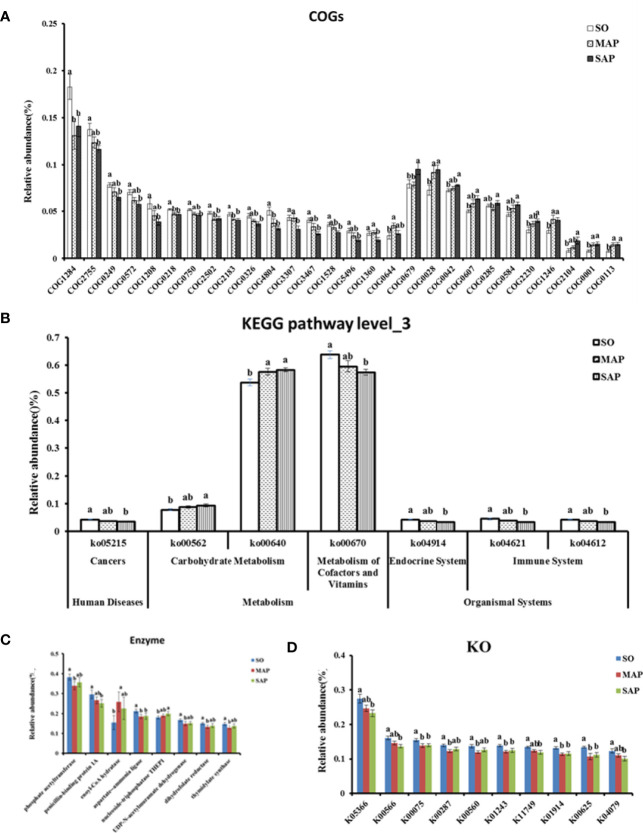
Significant difference in function predicted for COGs **(A)**, KEGG pathway level_3 **(B)**, Enzyme **(C)**, and KO **(D)**. SO, Sham operation; MAP, Mild acute pancreatitis; SAP, Severe acute pancreatitis; COG, Cluster of orthologous genes; KEGG, Kyoto Encyclopedia of Genes and Genomes; KO, KEGG orthology. Data are shown as mean ± standard error. Different letters (a, b) represent significant differences between three groups at P < 0.05.

The results of the COG functional annotation showed that the groups had functional differences in terms of 28 COGs (**Figure 7A**). Among them, five COGs relative abundance in AP group were significantly lower than that in SO group(*P* < 0.05). These COGs were related to the membrane (COG1284), which is necessary for normal cell division; maintenance of normal septation (by similarity) (COG0218); asparagine synthetase A (COG2502); transcriptional accessory protein (COG2183); and uncharacterized conserved protein (COG4804). Four COGs showed a contrasting trend (*P* < 0.05). These COGs were related to acetolactate synthase (COG0028); N-acetyl-glutamate synthase (COG1246); glutamate-1-semialdehyde aminotransferase (COG0001); and delta-aminolevulinic acid dehydratase (COG0113). Four COGs had significant differences in abundance between MAP group and SAP group (*P* < 0.05). They were related to O-antigen polymerase (COG3307), OmpA/MotB domain protein (COG1360), auxin efflux carrier (COG0679), and folylpolyglutamate synthase (COG0285). One COG in MAP group and fourteen COGs in SAP group respectively were significantly different from that in SO group (*P* < 0.05).

According to the KEGG database, we found seven Level_3 pathways that were significantly different among the three groups (*P* < 0.05) (**Figure 7B**). Among them, propanoate metabolism (Ko00640) in AP group was significantly higher than that in SO group (*P* < 0.05). Others were only found significantly difference between SAP and SO group (*P* < 0.05). Eight enzymes differed significantly among the three groups (*P* < 0.05) (**Figure 7C**). The aspartate‒ammonia ligase in the AP group was lower than that in the SO group (*P* < 0.05). Five enzymes in MAP group and two enzymes in SAP group respectively were significantly different from that in SO group (*P* < 0.05).

Ten KEGG orthology (KO) groups were significantly different among the three groups (*P* < 0.05) (**Figure 7D**). Four KOs in the AP group were lower than those in the SO group. Their functions were related to UDP-N-acetyl-muramate dehydrogenase (K00075), adenosylhomocysteine nucleosidase (K01243), aspartate–ammonia ligase (K01914), and phosphate acetyltransferase (K00625). Two KOs in MAP group and four KOs in SAP group respectively were significantly different from that in SO group (*P* < 0.05).

## Discussion

In this study, we investigated the succession of intestinal microbiota community structure and function during the early 72 hours of AP rats. The F/B ratio in SO group was significantly different from the group that in AP group at the phylum level. The relative abundance of *Lactobacillus* was significantly decreased from 24 h to 72 h in SAP group. Significant enrichment in microbial groups was found in comparison of SO and AP, and from comparing MAP with SAP, according to LEfSe analysis. The *Lactobacillus* was correlated with most COGs, compared to other genus. Significant functional differences were also observed among the three groups. These results suggest that some community succession character may be used as potential biomarker to distinguish MAP from SAP rats at the initial stages.

Bacterial community differences were detected at 24 h among the groups; the most significant succession was found at 72 h in the SAP group. This result is similar to several previous studies. For example, the intestinal microbial community has been reported to differ between AP patients (prior to treatment) and healthy volunteers (Zhang et al., 2018). Moreover, gut microbiome dysbiosis worsens the severity of AP in mice at 24 h after induction (Zhu et al., 2019).

The dominant phylum was *Firmicute*s, followed by *Bacteroidetes*. There was a marked difference in the F/B ratio between the SO group and AP group. In particular, at 72 h, the F/B ratio of the SAP group had an approximately 1.7-fold increase, while that in the MAP group showed a nearly 5-fold decrease as compared to the SO group (**Figure 2B**). These results suggest that the F/B ratio could be a potential marker for the classification of MAP and SAP relative to individuals without AP. This is consistent with previous studies on several other diseases. For instance, the microbiota of patients with irritable bowel syndrome showed a 2-fold increase in the F/B ratio compared to controls (Rajilić–Stojanović et al., 2011). An increased F/B ratio was also detected in obese individuals (Koliada et al., 2017), in spontaneously hypertensive rats (Yang et al., 2015), and in patients with coronary artery disease (CAD) (Yamashita et al., 2016), autism (Strati et al., 2017), and HBV-related liver cirrhosis (Deng et al., 2019). These results support the F/B ratio increase that we observed in the SAP group and suggest that these diseases may have some common features. Furthermore, an increased F/B ratio may be a cause of SAP and a predictor of future complications. In addition, the F/B ratio was found to be decreased in patients with type 1 diabetes (Murri et al., 2013; Demirci et al., 2020), similar to our observations in the MAP group. These results suggest that the F/B ratio is a powerful biomarker in the initial stages of AP development. However, the human microbiota F/B ratio changes with age and sex (Mariat et al., 2009; Salah et al., 2019), and this should be considered in further research, particularly in AP patients.

According to LEfSe analysis, the most enriched genus in the AP groups, compared to the SO group, was the family *Clostridiaceae* 1 and genus *Clostridium sensu stricto* 1 (**Figure 5A**). This result indicates that these two groups of microbes may be biomarkers of AP. Further research is needed to clarify the role of these two groups of opportunistic pathogens. Species of the genus *Clostridium* represent a double-edged sword. For example, *Clostridium perfringens* was an important biomarker of necrotizing pancreatitis (Biswas et al., 2017). *Clostridium lituseburense-*like bacteria have been reported as “AP-associated microbiota” (Gerritsen et al., 2011). However, *Clostridium butyricum* strains suppress experimental AP by maintaining intestinal homeostasis (Pan et al., 2019). Furthermore, the genus *Blautia* in the SAP group has significantly higher relative abundance compared to the SO group (*P* < 0.05). It has been reported that increased *Blautia* abundance is associated with a reduction in the lethality of graft-versus-host disease (Jenq et al., 2015). This result suggests that the physiological commensal/pathogen ratio was significantly altered in AP rats.

Differences in bacterial community structure and predictive function were found between the MAP and SAP groups. LEfSe analysis showed that the genera *Collinsella*, *Morganella*, and *Blautia* were enriched in the MAP group, compared to those in the SAP group. In particular, the *Bacteroidales* DS24-7 groups were enriched in the SAP group (**Figure 5B**). At 72 h, the relative abundance of K was significantly lower in the MAP group, compared to the SAP group, while the relative abundance of J showed the opposite pattern (*P* < 0.05). *Romboutsia* abundance was significantly correlated with K, indicating that the SAP and MAP rats had different bacterial communities, which may be involved in different functions. This inference is supported by other studies. For example, although the amount of *Bifidobacteria*, *Lactobacillus*, and *Clostridium leptum* decreased, the amount of *Bacillus*, *galactococcus*, and *Stenotrophomonas* was higher in SAP patients compared to MAP patients (Cen et al., 2018). *Enterococcus* was increased, and *Bifidobacterium* was decreased in patients with SAP compared with patients with MAP (Tan et al., 2015). These results demonstrate that the significant enriched bacterial group at different time of AP onset may be not the same.

*Lactobacillus* was the most abundant genus and may perform a key role in AP. At 48 h, the relative abundance of *Lactobacillus* in the SAP group was significantly higher than in the SO group (*P* < 0.05). This result was similar to that in type-2 diabetes patients (Karlsson et al., 2013; Sato et al., 2014). However, at 72 h, the relative abundance of *Lactobacillus* nearly disappeared in the SAP group. In contrast, it was detected in the MAP group (**Figure 2** and **Figure S3**). *Lactobacillus* was significantly related to nine COGs (**Tables S1, S2**); once its abundance decreased sharply, these COGs would also be decreased or eliminated, resulting in significant changes in intestinal physiological function. Above all, the relative abundance of *Lactobacillus* was a very important feature in SAP progression. In theory, timely supplementation with *Lactobacillus* could return intestinal physiological function to normal. This hypothesis is supported by a few previous studies. For example, enteral feeding with *Lactobacillus plantarum* attenuates disease severity, decreases intestinal permeability, and improves clinical outcomes in patients with AP (Qin et al., 2008). *Lactobacillus* can improve physiological function and cognitive ability in aged mice by regulating the gut microbiome (Ni et al., 2019). These results suggest that the genus *Lactobacillus* should be a potential therapeutic target, and the time period from 48 h to 72 h might be critical for AP treatment.

Through functional prediction, some COG functions and KEGG pathways were found decreased or increased in the AP group, compared to the SO group (**Figure 7**). This result suggests that the intestinal microbiome function was significantly different in the two groups. COGs also showed significant correlations between different genera (**Tables S1, S2**). This result supports the view that species–function relationships shape host gut microbiome ecological properties (Vieira-Silva et al., 2016). Some functions were also detected significant difference between MAP group and SAP group. These results suggest that the intestinal microbial functional changes in AP groups of rats were complex, further research were needed to reveal the deeper mechanisms of them in AP development.

Despite the novel findings of this study, there were still some limitations. We summarize the limitations of this study as follows (1): The sample was used only animal models, human patients were not included (2), The results would be strengthened by adding further analysis of characteristic metabolites in feces; and (3) In future studies, the transcriptomics and sequencing combined with metabolomics should be implemented to determine functional implications.

## Conclusions

In summary, the gut microbiome structure significantly differed between AP group and SO group during the initial 72 hours. At the phylum level, *Firmicutes* was dominant, and its relative abundance increased from 24 h to 72 h in the three groups. Compared to the SO group, the F/B ratio was significantly increased in the SAP group and decreased in the MAP group, particularly at 72 h (*P* < 0.05). *Lactobacillus* was the most abundant genus, and its relative abundance in the SAP group was significantly decreased at 72 h. By LEfSe analysis, the most enriched bacteria in the AP group relative to the SO group was the family *Clostridiaceae* 1 and genus *Clostridium sensu stricto* 1. The relative abundance of the genera *Collinsella*, *Morganella*, and *Blautia* in the MAP group was enriched as compared to the SAP group; the genus *Bacteroidales* DS24-7 was more enriched in the SAP group than in the MAP group (*P* < 0.05). COG and KEGG analysis, based on PICRUSt, also showed that the sequences with relative abundance differed markedly among the three groups. Thirteen genera were significantly correlated with COG function. *Lactobacillus* was significantly correlated with the most COGs (nine). Some predicted COGs and KEGG pathways differed significantly among the three groups, indicating functional differences between the AP and SO groups. These results suggest that the F/B ratio and the relative abundance of *Lactobacillus* are potential markers for distinguishing between the SAP and MAP groups.

## Data Availability Statement

The datasets presented in this study can be found in online repositories. The names of the repository/repositories and accession number(s) can be found below: NCBI [accession: PRJNA602368].

## Ethics Statement

The animal study was reviewed and approved by Experimental Animal Ethics Committee of Southwest Medical University.

## Author Contributions

JL and XX conceived the experiment. JL and ML carried out the experiments and investigation. SQ and BL provided the resources. JL and LH wrote the original draft. LH and XX wrote the review and edited the paper. All authors contributed to the article and approved the submitted version.

## Funding

This work was supported by the Luzhou-Southwest Medical University Joint Project (2017LZXNYD-J31 and 2017LZXNYD-T11), Southwest Medical University Youth Project (2017-ZRQN-078 and 2017-ZRZD-004), and the Affiliated Hospital Youth Project of Southwest Medical University (2017-QB-2 and 2017-QB-3).

## Conflict of Interest

The authors declare that the research was conducted in the absence of any commercial or financial relationships that could be construed as a potential conflict of interest.

## Publisher’s Note

All claims expressed in this article are solely those of the authors and do not necessarily represent those of their affiliated organizations, or those of the publisher, the editors and the reviewers. Any product that may be evaluated in this article, or claim that may be made by its manufacturer, is not guaranteed or endorsed by the publisher.
