# Supplementary material for: Significant Succession of Intestinal Bacterial Community and Function During the Initial 72 Hours of Acute Pancreatitis in Rats
Source: Front Cell Infect Microbiol. 2022 Apr 29;12:808991. doi: 10.3389/fcimb.2022.808991 (PMC9105020; doi:10.3389/fcimb.2022.808991)

**Supplementary materials**

Table.S1. The genus correlated with abundant COGs which relative abundance was ≥5%.

| COG | Related species | R^2^ | P value |
| --- | --- | --- | --- |
| E Amino acid transport and metabolism | Escherichia-Shigella | .733* | 0.025 |
|  | norank_f__Erysipelotrichaceae | .667* | 0.05 |
| R General function prediction only | Lactobacillus | -.717* | 0.03 |
|  | Allobaculum | .667* | 0.05 |
|  | Turicibacter | .850** | 0.004 |
|  | Blautia | .717* | 0.03 |
| S Function unknown | Escherichia-Shigella | .700* | 0.036 |
|  | unclassified_f__Lachnospiraceae | -.700* | 0.036 |
| J Translation, ribosomal structure and biogenesis | norank_f__Bacteroidales_S24-7_group | .667* | 0.05 |
|  | Prevotellaceae_UCG-003 | **.917**** | 0.001 |
| M Cell wall/membrane/envelope biogenesis | norank_f__Bacteroidales_S24-7_group | .767* | 0.016 |
|  | unclassified_f__Lachnospiraceae | **-.933**** | 0 |
|  | Ruminococcaceae_UCG-014 | -.833** | 0.005 |
|  | Candidatus_Saccharimonas | -.833** | 0.005 |
| K Transcription | Romboutsia | .850** | 0.004 |
|  | unclassified_f__Lachnospiraceae | .867** | 0.002 |
|  | Candidatus_Saccharimonas | .783* | 0.013 |
|  | Blautia | .667* | 0.05 |
| C Energy production and conversion | Prevotellaceae_UCG-003 | -.750* | 0.02 |
| P Inorganic ion transport and metabolism | Lactobacillus | .733* | 0.025 |
|  | Allobaculum | -.733* | 0.025 |
|  | Romboutsia | **-.950**** | 0 |
|  | Escherichia-Shigella | .800** | 0.01 |
|  | unclassified_f__Lachnospiraceae | -.667* | 0.05 |
|  | Candidatus_Saccharimonas | -.683* | 0.042 |
| T Signal transduction mechanisms | Romboutsia | .683* | 0.042 |
|  | norank_f__Bacteroidales_S24-7_group | -.717* | 0.03 |
|  | unclassified_f__Lachnospiraceae | **1.000**** | . |
|  | Ruminococcaceae_UCG-014 | .750* | 0.02 |
|  | Candidatus_Saccharimonas | .850** | 0.004 |

Note: * Significant correlation at *P* < 0.05; ****** Significant correlation at *P* < 0.01.

Table.S2. The genus correlated with abundant COGs which relative abundance was 0.8% - 5%.

| COG | Related species | R^2^ | P value |
| --- | --- | --- | --- |
| O Posttranslational modification, protein turnover, chaperones | Romboutsia | -.883** | 0.002 |
|  | Escherichia-Shigella | .750* | 0.02 |
|  | unclassified_f__Lachnospiraceae | -.867** | 0.002 |
|  | Ruminococcaceae_UCG-014 | -.717* | 0.03 |
|  | Candidatus_Saccharimonas | -.800** | 0.01 |
| F Nucleotide transport and metabolism | Clostridium_sensu_stricto_1 | -.850** | 0.004 |
|  | norank_f__Bacteroidales_S24-7_group | .683* | 0.042 |
|  | Prevotellaceae_UCG-003 | .683* | 0.042 |
| H Coenzyme transport and metabolism | Lactobacillus | .667* | 0.05 |
|  | Romboutsia | -.750* | 0.02 |
|  | Escherichia-Shigella | .667* | 0.05 |
| V Defense mechanisms | Lactobacillus | -.883** | 0.002 |
|  | Allobaculum | .733* | 0.025 |
|  | Romboutsia | .800** | 0.01 |
|  | Escherichia-Shigella | -.717* | 0.03 |
|  | Turicibacter | .733* | 0.025 |
| I Lipid transport and metabolism | Lactobacillus | .750* | 0.02 |
|  | Allobaculum | -.867** | 0.002 |
|  | Turicibacter | -.683* | 0.042 |
| U Intracellular trafficking, secretion, and vesicular transport | Lactobacillus | .783* | 0.013 |
|  | Allobaculum | -.683* | 0.042 |
|  | Romboutsia | **-.917**** | 0.001 |
|  | Escherichia-Shigella | .767* | 0.016 |
|  | Turicibacter | -.683* | 0.042 |
|  | unclassified_f__Lachnospiraceae | -.700* | 0.036 |
| D Cell cycle control, cell division, chromosome partitioning | Lactobacillus | -.817** | 0.007 |
|  | Allobaculum | .667* | 0.05 |
|  | Romboutsia | .850** | 0.004 |
|  | Escherichia-Shigella | -.717* | 0.03 |
|  | Turicibacter | .767* | 0.016 |
|  | unclassified_f__Lachnospiraceae | .667* | 0.05 |
| Q Secondary metabolites biosynthesis, transport and catabolism | Lactobacillus | .800** | 0.01 |
|  | Allobaculum | -.867** | 0.002 |
|  | Prevotellaceae_UCG-003 | -.667* | 0.05 |
| N Cell motility | Lactobacillus | .800** | 0.01 |
|  | Allobaculum | -.750* | 0.02 |
|  | Turicibacter | -.717* | 0.03 |
|  | Prevotellaceae_UCG-003 | -.817** | 0.007 |

Note: * Significant correlation at *P* < 0.05; ****** Significant correlation at *P* < 0.01.

Fig.S1 OTUs analysis in AP rats intestinal farces sample showed by Venn diagrams. (A) The common or unique OTUs in three groups; (B)-(D): From 24h to 72h, the common or unique OTUs at three times in the same group. The abbreviations SO, MAP and SAP were present for sham operation, mild acute pancreatitis, and severe acute pancreatitis, respectively.


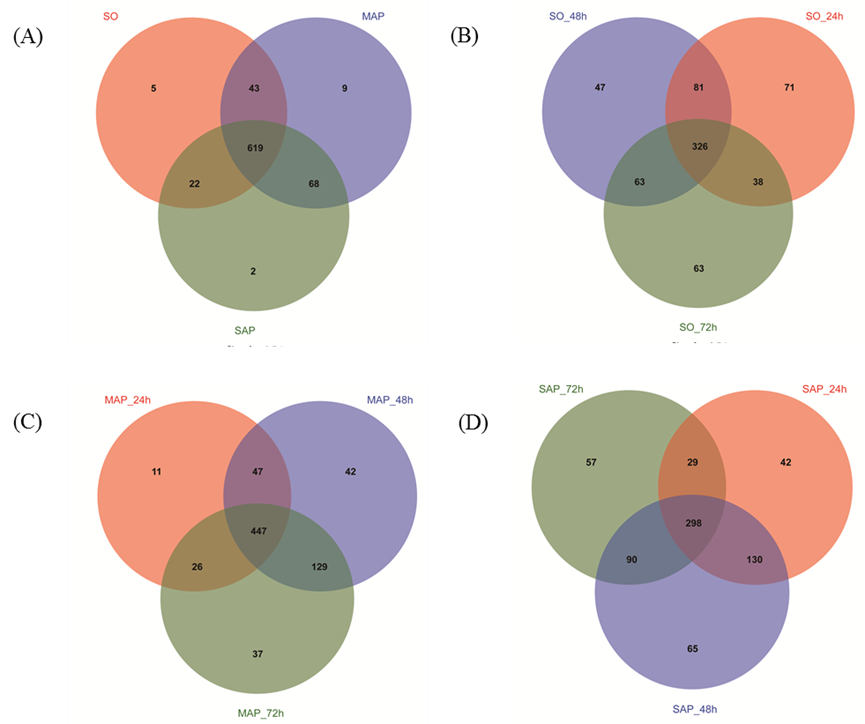


Fig. S2 Eleven abundant COGs succession from 24h to 72h in three groups. The abbreviations SO, MAP and SAP were present for sham operation, mild acute pancreatitis, and severe acute pancreatitis, respectively.


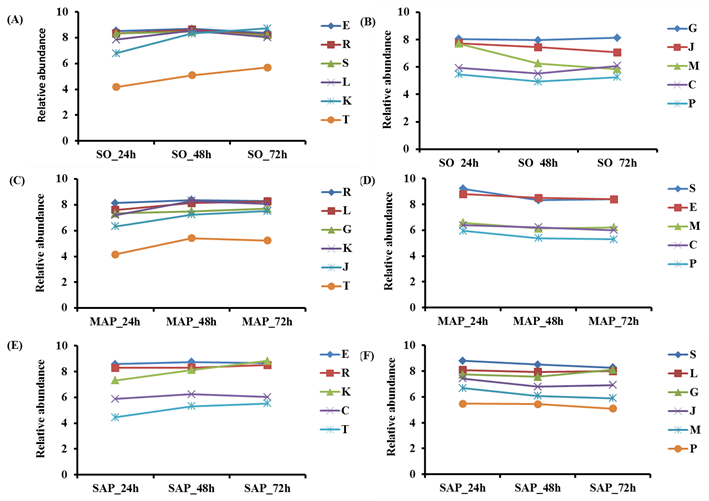


Fig. S3 The abundant genus in phylum Firmicutes (A) and Bacteroidetes (B), and the ratio of total relative abundance of the abundant genus in Firmicutes/Bacteroidetes (F/B) in three groups (C). The abbreviations SO, MAP and SAP were present for sham operation, mild acute pancreatitis, and severe acute pancreatitis, respectively.

 
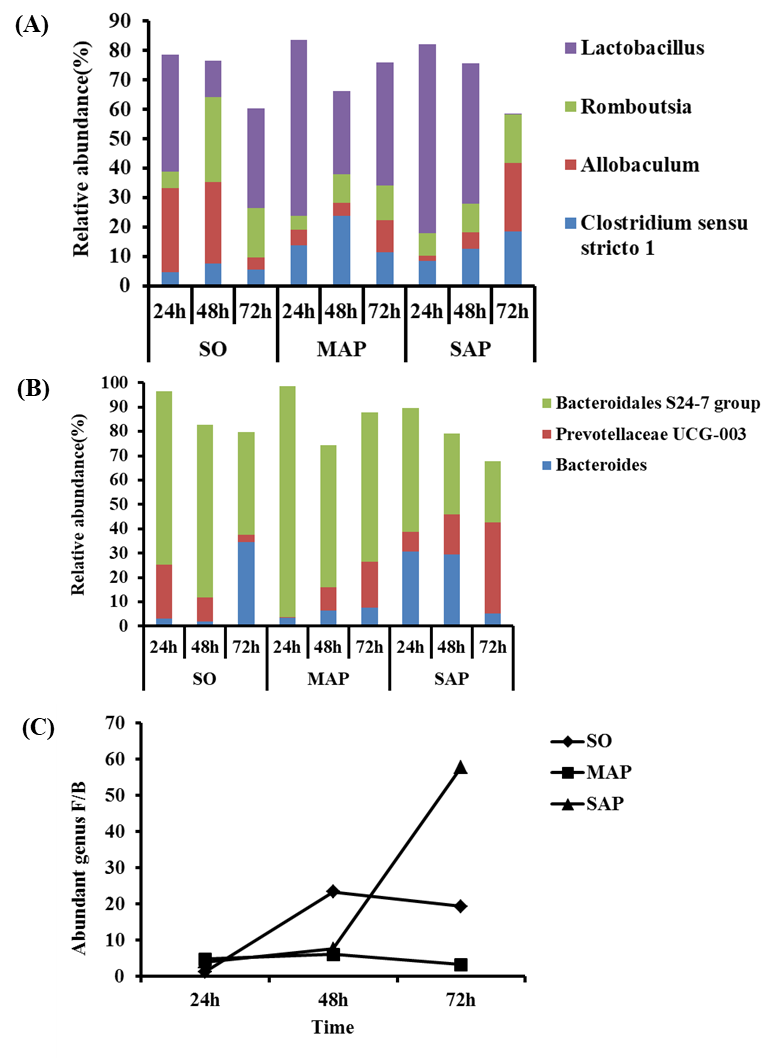

Supplement: Supplementary file 1 [file DataSheet_1.docx]
